# COMPARISON OF MULTIDIMENSIONAL AGING TRAJECTORIES BETWEEN PEOPLE AGING WITH AND WITHOUT DISABILITIES

**DOI:** 10.1093/geroni/igae098.4296

**Published:** 2024-12-31

**Authors:** Seeun Park, Lisa Bratzke

**Affiliations:** University of Washington, Seattle, Washington, United States; University of Wisconsin-Madison, University of Wisconsin-Madison, Wisconsin, United States

## Abstract

**Background:**

The aging trajectories of people aging with disabilities (PAwD) may involve distinct life experiences compared to those of people without disabilities (PWoD). However, the extent to which aging trajectories vary between the two groups remains unclear. This study investigates these differences, analyzing the impact of long-term disability at phenotypic and functional levels, and examining social disparities across various demographic factors using data from the 2006-2020 waves of the Health and Retirement Study.

**Methods:**

Phenotypic aging was measured by the metabolic dysfunction risk score (MDRS), and functional aging was assessed through limitations in activities of daily living (ADLs) and instrumental activities of daily living (IADLs). Growth-curve modeling was used.

**Results:**

A total of 7,234 participants were included. PAwD exhibited significantly higher baseline MDRS and ADLs/IADLs scores, with more rapid escalation over time, indicating premature and accelerated aging. Significant social disparities in aging were observed across cohort, gender, race/ethnicity, and educational attainment. Recent cohorts of PAwD showed more rapid increases in MDRS. Women and racial minorities, especially non-Hispanic Blacks and Hispanics, had higher MDRS. Across all race/ethnicity groups, PAwD exhibited higher MDRS and functional limitations. Lower educational attainment was associated with both higher MDRS and ADLs/IADLs.

**Conclusion:**

PAwD experience an accelerated and unequal aging process compared to PWoD, with significant social disparities. These findings suggest the need for tailored health interventions and policies that address the distinct aging trajectories and vulnerabilities of PAwD, emphasizing early detection and targeted support to mitigate the compounded effects of disability and sociodemographic factors.

